# Persistence of Infectious Canine Distemper Virus in Murine Xenotransplants of Canine Histiocytic Sarcoma Cells after Intratumoral Application

**DOI:** 10.3390/ijms25158297

**Published:** 2024-07-30

**Authors:** Mara Sophie Lombardo, Federico Armando, Katarzyna Marek, Karl Rohn, Wolfgang Baumgärtner, Christina Puff

**Affiliations:** 1 Department of Pathology, University of Veterinary Medicine Hannover, 30559 Hannover, Germany; mara.sophie.lombardo@tiho-hannover.de (M.S.L.); federico.armando@unipr.it (F.A.); wolfgang.baumgaertner@tiho-hannover.de (W.B.); 2 Pathology Unit, Veterinary Medicine Department, University of Parma, 43126 Parma, Italy; 3 Institute for Biometry, Epidemiology and Information Processing, University of Veterinary Medicine Hannover, 30559 Hannover, Germany; karl.rohn@tiho-hannover.de

**Keywords:** canine distemper virus, canine histiocytic sarcoma, virus persistence

## Abstract

Oncolytic viruses and morbilliviruses in particular, represent an interesting therapeutic approach for tumors with a poor prognosis and frequent resistance to conventional therapies. Canine histiocytic sarcomas (HS) exemplify such a neoplasm in need for new curative approaches. Previous investigations demonstrated a limited success of an acute intratumoral application of canine distemper virus (CDV) on xenotransplanted canine histiocytic sarcoma cells (DH82 cells), while persistently CDV-infected DH82 cell transplants exhibited a complete spontaneous regression. Therefore, the present study focuses on an intratumoral application of persistently CDV vaccine strain Onderstepoort-infected DH82 (DH82 Ond p.i.) cells into non-infected subcutaneous DH82 cell transplants in a murine model. DH82 cell transplants that received 10 applications, two days apart, showed a transient growth retardation as well as larger areas of intratumoral necrosis, lower mitotic rates, and a decreased intratumoral vascularization compared to controls. Viral mRNA was detected in all neoplasms following application of DH82 Ond p.i. cells until 66 days after the last injection. Furthermore, infectious virus was present until 62 days after the last injection. Although complete regression was not achieved, the present application regimen provides promising results as a basis for further treatments, particularly with genetically modified viruses, to enhance the observed effects.

## 1. Introduction

Histiocytic sarcomas originate from macrophages or dendritic cells [1,2] and show a higher incidence in dogs compared to cats and humans [2,3]. There are some dog breeds, including Bernese mountain dogs, Rottweilers, and Golden and Flat coated Retrievers, which show a higher incidence of histiocytic sarcomas [3,4]. Histiocytic sarcomas are known to occur in a localized form, often present in the periarticular subcutis and, occasionally, in the central nervous system, or as a disseminated disease with spleen, bone marrow, lymph nodes, lungs, or liver as the commonly affected organs. Macroscopically, the tumors present as firm, white to tan masses with a uniform appearance [4]. Histolo-gically, pleomorphic, often multinucleated, giant cells are frequently observed. Often, the neoplastic cells show a high degree of pleomorphism and bizarre mitotic figures [4]. Disseminated histiocytic sarcomas have a poor prognosis, which can be improved only partially by conventional chemo- or radiotherapy [5]. Affected dogs have a median survival time of less than one year, even when treated [6].

An alternative for or an addition to these conventional therapies could be the use of oncolytic viruses. Oncolytic viruses have been discovered rather accidentally by observing a tumor regression after a spontaneous infection or after vaccinations. These first observations dated back to the early 20th century [7,8,9] and, since then, have been studied intensively. Tumor regression by oncolytic viruses is not only achieved by direct cell lysis but also by modulation of the tumor microenvironment [10,11,12]. For example, it has been shown that viral infections of neoplasms lead to a decreased microvessel density [13] and, thus, to an intratumoral hypoxia [14]. Nowadays, there are not only naturally occurring wild-type viruses being used for therapeutic purposes but also genetically modified strains [15]. These modifications are used to increase cellular selectivity [12] or to function as a carrier for oncolytic agents [16,17,18]. In addition to an increased oncolytic effect, genetic engineering of oncolytic viruses may also lead to enhanced safety [7].

Today, there are approved oncolytic viruses in human medicine already available, including a modified adenovirus (Oncorine, H101), which was approved in China in 2006 [19]. Furthermore, there are also other promising candidates, namely vaccinia virus [15], herpes simplex virus [15], and measles virus [20]. The latter is a Morbillivirus and belongs to the family Paramyxoviridae. Thus, it is closely related to canine distemper virus (CDV). This enveloped, single-stranded, negative sense RNA virus is able to infect many different cell types, including monocytes and macrophages [21,22].

DH82 cells are a permanent cell line, isolated from the bone marrow of a dog suffering from a disseminated histiocytic sarcoma. These cells are, nowadays, broadly used for research purposes [23,24,25,26]. Furthermore, an attenuated vaccine strain of CDV (strain Onderstepoort, CDV-Ond) was used for the generation of persistently CDV-infected DH82 cells (DH82 Ond p.i.) [26]. Previous studies using a murine DH82 xenotransplantation model demonstrated a reduced tumor growth after acute intratumoral infection with CDV-Ond [27], whereas a transplantation of DH82 Ond p.i. cells resulted in a complete spontaneous regression after an initial tumor formation [13]. Unfortunately, the latter model does not reflect clinical circumstances.

In the present study, a more clinically relevant approach to achieve a permanent intratumoral virus source was chosen. Therefore, the aim of this study was to investigate the influence of an intratumoral application of DH82 Ond p.i. cells in established, subcutaneously xenotransplanted canine histiocytic sarcoma cells in a murine model. Special emphasis was given to the presence of infectious virus within the neoplasms at different time points after intratumoral DH82 Ond p.i. cell application.

## 2. Results

### 2.1. Relative Tumor Growth

**Figure 1:**
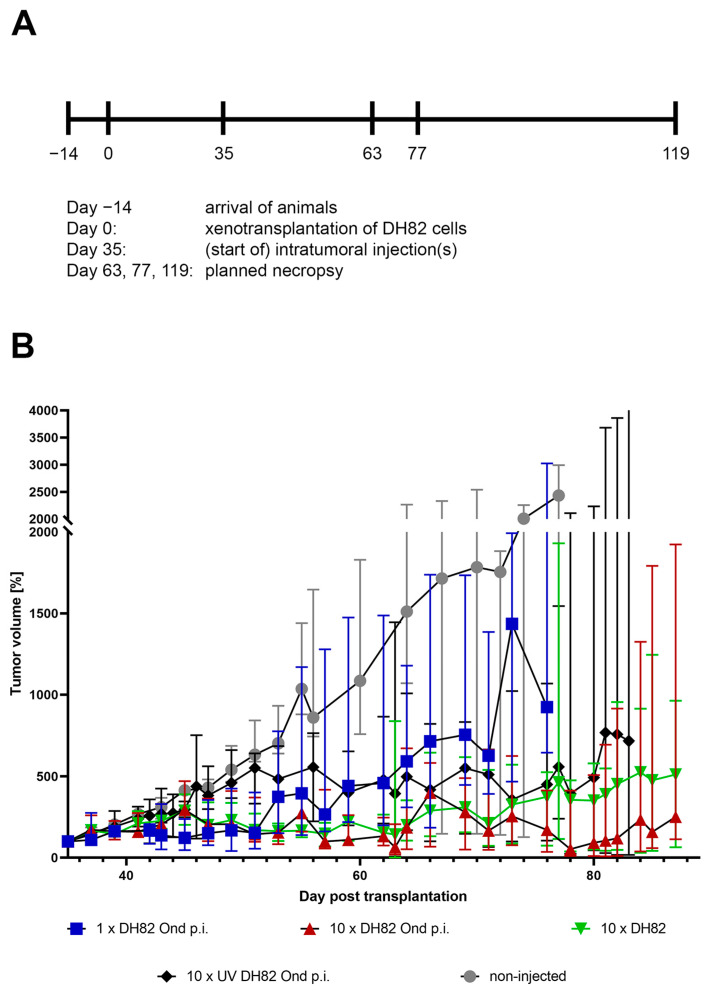
(**A**) Schematic time course of the animal experiment. (**B**) Development of the relative tumor volume (median, 95% confidence interval, trend lines). Default value (100%) was set immediately prior to the first intratumoral injection [35 days post transplantation (dpt)]. 1× DH82 Ond p.i. (blue): tumors after a single intratumoral injection of persistently canine distemper virus strain Onderstepoort-infected DH82 cells (DH82 Ond p.i.); 10× DH82 Ond p.i. (red): tumors after ten intratumoral injections of DH82 Ond p.i. cells; 10× DH82 (green): tumors after ten intratumoral injections of DH82 cells; 10× UV DH82 Ond p.i. (black): tumors after ten intratumoral injections of UV-inactivated, homogenized DH82 Ond p.i. cells; non-injected (grey): tumors that did not receive any injection. Each group consisted initially of 6 mice (n = 6) per time point and treatment. Please note that some animals had to be preliminarily sacrificed and did not reach the planned time point for necropsy.

For the calculation of the relative tumor growth, the tumor volumes at 35 days post-transplantation (dpt), immediately before the first intratumoral application of the persistently CDV vaccine strain Onderstepoort-infected DH82 (DH82 Ond p.i.) cells, were taken as the default value (100%). A pairwise comparison of the different groups [group 1: one intratumoral application with DH82 Ond p.i. cells (1× DH82 Ond p.i.); group 2: 10 intratumoral applications with DH82 Ond p.i. cells (10× DH82 Ond p.i.); group 3: 10 intratumoral applications with DH82 cells (10× DH82); group 4: 10 intratumoral applications with homogenized, UV-inactivated DH82 p.i. cells (10× UV DH82 Ond p.i.); group 5: non-injected tumors)] was performed every week, as long as there were at least four animals per group available (93 dpt for 10× DH82 Ond p.i., 89 dpt 10× DH82, and 86 dpt for 10× UV DH82 Ond p.i.). Animals were preliminary sacrificed when the tumors reached a mean diameter larger than 1.5 cm, if the tumors became ulcerated, or if the animal reached a certain score in a clinical examination, which included appearance, behavior, and tumor examination. For non-treated tumor-bearing animals and tumor development after a single injection of DH82 Ond p.i. cells, the data were only available until 77 dpt, according to the study design (Figure 1A). The results of the clinical investigation are displayed in Figure 1B. Table S1 gives an overview of the statistically significant differences between the groups at the different time points. Remarkably, the 10× DH82 Ond p.i. tumors showed a statistically smaller relative tumor volume than all other groups at 71 dpt and 76/77 dpt. Additionally, the same group presented with statistically smaller relative tumor volumes than all other groups except the 10× DH82 group at 56 dpt and 63 dpt. The 1× DH82 Ond p.i. tumors exhibited a smaller relative tumor volume than the non-injected tumors and 10× UV-DH82 Ond p.i. at 42 dpt, 49 dpt, and 63 dpt.

### 2.2. Histological and Immunohistological Results

The neoplasms were analyzed at different time points using histology and immunohistology. A pairwise comparison of the groups was performed at two time points (63 and 77 dpt). A third time point at 119 dpt was planned for the tenfold-injected groups. However, it was excluded from further analysis since there were less than four animals per group remaining because the other animals of this group had to be preliminarily sacrificed (see above). For the 1× DH82 Ond p.i. group, there was only one time point available (77 dpt). An overview of all group medians as well as minimum and maximum values per group of all histological and immunohistological parameters is given in Table S2 of the Supplementary Material.

#### 2.2.1. Necrosis

**Figure 2:**
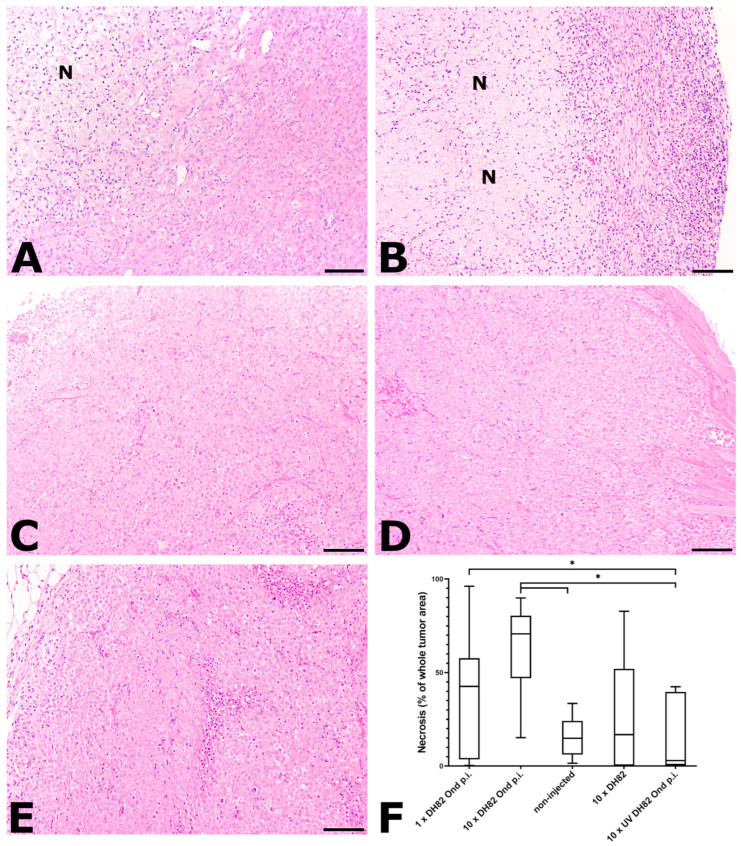
Tumor morphology and percentage of necrosis at 77 dpt. Necrosis was defined as hyper-eosinophilic area with loss of cellular details, karyorrhexis, karyopyknosis, and karyolysis and accumulations of cellular debris (N). Each group consisted initially of 6 mice (n = 6) per time point and treatment. Please note that some animals had to be preliminarily sacrificed and did not reach the planned time point for necropsy. (**A**) 1× DH82 Ond p.i. neoplasms show a moderate percentage of necrosis; (**B**) 10× DH82 Ond p.i. tumors display large areas of necrosis; (**C**) Non-injected tumors; (**D**) 10× DH82 neoplasms; and (**E**) 10× UV DH82 Ond p.i. tumors show only small areas of necrosis. (**F**) Graphical display of the percentage area of necrosis in the different groups. 1× DH82 Ond p.i.: tumors after a single intratumoral injection (1× DH82 Ond p.i.) of persistently canine distemper virus strain Onderstepoort-infected DH82 cells (n = 6); 10× DH82 Ond p.i.: tumors after ten intratumoral injections of DH82 Ond p.i. cells (n = 6); 10× DH82: tumors after ten intratumoral injections of DH82 cells (n = 6); 10× UV DH82 Ond p.i.: tumors after ten intratumoral injections of UV-inactivated, homogenized DH82 Ond p.i. cells (n = 5); non-injected: tumors that did not receive any injection (n = 5). * *p* < 0.05. Scale bar: 100 µm; hematoxylin–eosin.

Necrosis was defined as hypereosinophilia with loss of cellular details, karyorrhexis, karyopyknosis, and karyolysis and accumulations of cellular debris (Figure 2). The 10× DH82 Ond p.i. group displayed the largest median necrotic area of all groups at both time points (29.19% at 63 dpt; 70.71% at 77 dpt). The smallest median necrotic area at 63 dpt was observed in the non-injected tumors (0.49%) and at 77 dpt in the 10× UV-DH82 Ond p.i. group (4.76%). At 63 dpt, the 10× DH82 Ond p.i. tumors showed a significantly higher percentage of necrosis than the non-injected tumors and 10× DH82 tumors. Additionally, at this time point, the non-injected tumors presented with a significantly smaller percentage of necrosis than the 10× UV-DH82 Ond p.i. group. At 77 dpt, the percentage area of necrosis was significantly larger in 10× DH82 Ond p.i. than in the non-injected tumors and in the 10× UV-DH82 Ond p.i. tumors. In addition, the latter displayed a significantly smaller area of necrosis than the 1× DH82 Ond p.i. tumors at this time point (Figure 2F).

#### 2.2.2. Intratumoral Apoptotic Rate

**Figure 3:**
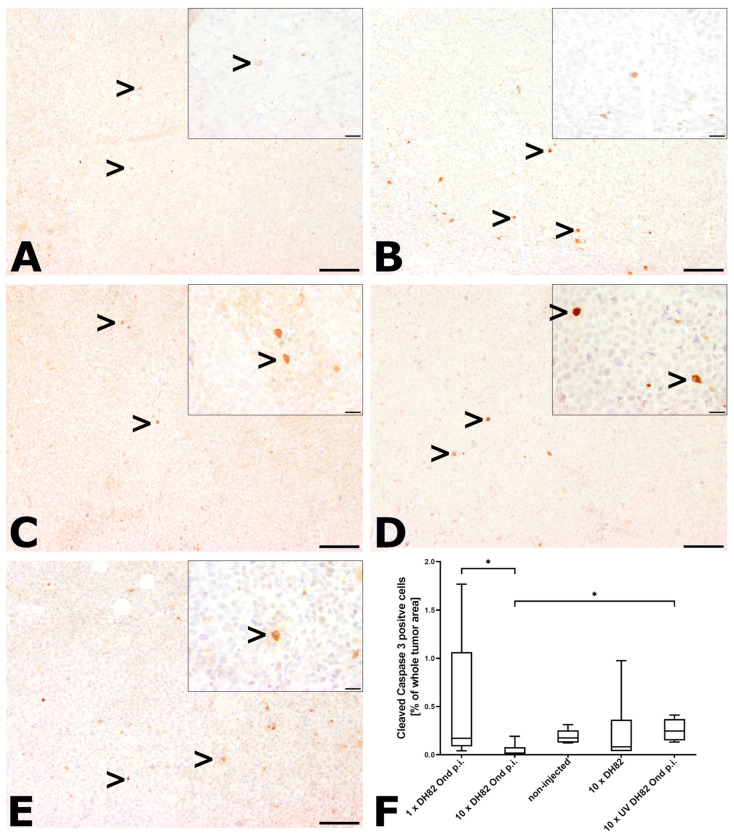
Cleaved caspase 3 immunolabeling at 77 dpt. Positive cells show a distinct granular to coarse cytoplasmic staining (>). There were only single positive cells observed in the tumors of all groups. (**A**) 1× DH82 Ond p.i. tumor; (**B**) 10× DH82 Ond p.i. neoplasm; (**C**) non-injected tumor; (**D**) 10× DH82 neoplasm; (**E**) 10× UV DH82 Ond p.i. tumor. (**F**) Graphical display of the percentage of cleaved caspase 3 positive cells in the different groups. 1× DH82 Ond p.i.: tumors after a single intratumoral injection (1× DH82 Ond p.i.) of persistently canine distemper virus strain Onderstepoort-infected DH82 cells (n = 6); 10× DH82 Ond p.i.: tumors after ten intratumoral injections of DH82 Ond p.i. cells (n = 6); 10× DH82: tumors after ten intratumoral injections of DH82 cells (n = 6); 10× UV DH82 Ond p.i.: tumors after ten intratumoral injections of UV-inactivated, homogenized DH82 Ond p.i. cells (n = 5); non-injected: tumors that did not receive any injection (n = 5). * *p* < 0.05. Scale bar: 100 µm; scale bar of insert: 20 µm.

For the determination of the apoptotic rate, slides were stained with an anti-cleaved caspase 3 antibody, and the relative amount of intratumoral cells with a distinct granular to coarse cytoplasmic immunoreactivity was determined (Figure 3). Necrotic areas were excluded from the analysis. In all groups and time points, only two animals showed values higher than 1%. At both time points, the 10× DH82 Ond p.i. neoplasms exhibited the smallest median percentage of apoptotic cells (0.11% at 63 dpt; 0.02% at 77 dpt). The highest median of apoptotic cells at 63 dpt was found in the10× DH82 group (0.2%) and at 77 dpt in the 10× UV-DH82 Ond p.i. tumors (0.25%). At 63 dpt, the non-injected controls exhibited a significantly higher apoptotic rate than the 10× DH82 tumors. At 77 dpt, the 10× DH82 Ond p.i. tumors presented with a lower apoptotic rate than the 1× DH82 Ond p.i. and 10× UV DH82 Ond p.i. groups (Figure 3E).

#### 2.2.3. Mitotic Rate

**Figure 4:**
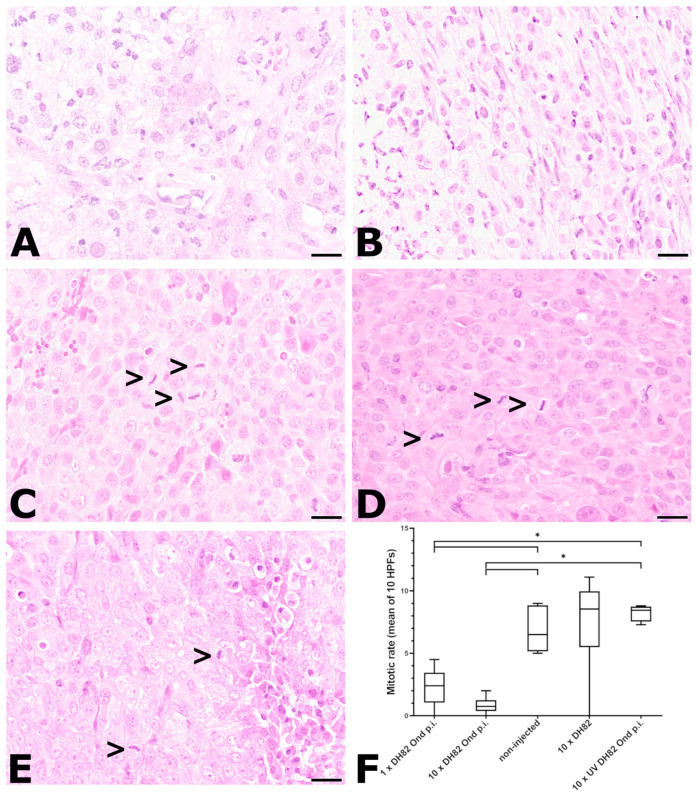
Mitotic rate at 77 dpt. (**A**) 1× DH82 Ond p.i. tumors rarely showed mitotic figures. (**B**) Similarly, 10× DH82 Ond p.i. neoplasms demonstrated only few mitotic figures. (**C**) Non-injected tumors, (**D**) 10× DH82 neoplasms, and (**E**) 10× DH82 UV Ond p.i. tumors display multiple mitotic figures (>). (**F**) Graphical display of the mitotic rate in the different groups. 1× DH82 Ond p.i.: tumors after a single intratumoral injection (1× DH82 Ond p.i.) of persistently canine distemper virus strain Onderstepoort-infected DH82 cells (n = 6); 10× DH82 Ond p.i.: tumors after ten intratumoral injections of DH82 Ond p.i. cells (n = 6); 10× DH82: tumors after ten intratumoral injections of DH82 cells (n = 6); 10× UV DH82 Ond p.i.: tumors after ten intratumoral injections of UV-inactivated, homogenized DH82 Ond p.i. cells (n = 5); non-injected: tumors that did not receive any injection (n = 5). * *p* < 0.05. Scale bar: 50 µm; hematoxylin–eosin.

The proliferation of the neoplastic cells was assessed by determining the mitotic rate, which is displayed in Figure 4. The 10× UV-DH82 Ond p.i. tumors showed the lowest median number of mitotic figures at 63 dpt (0.2 mitotic figures/HPF). At 77 dpt, the lowest median number of mitotic figures was observed in the 10× DH82 Ond p.i. neoplasms (0.75 mitotic figures/HPF). The highest median number of mitoses was found in the 10 DH82 tumors at both time points (11.7 mitotic figures/HPF at 63 dpt; 8.55 mitotic figures/HPF at 77 dpt). At 63 dpt, the 10 DH82 neoplasms showed a significantly higher median number of mitotic figures than all other groups. Additionally, the non-injected tumors presented with a significantly higher mitotic rate than the 10× UV-DH82 Ond p.i. tumors at 63 dpt. At 77 dpt, the neoplasms injected once or ten times with DH82 Ond p.i. cells showed a significantly lower mitotic rate than the non-injected tumors and 10× UV-DH82 Ond p.i. tumors (Figure 4F).

#### 2.2.4. Intratumoral Vessel Density

**Figure 5:**
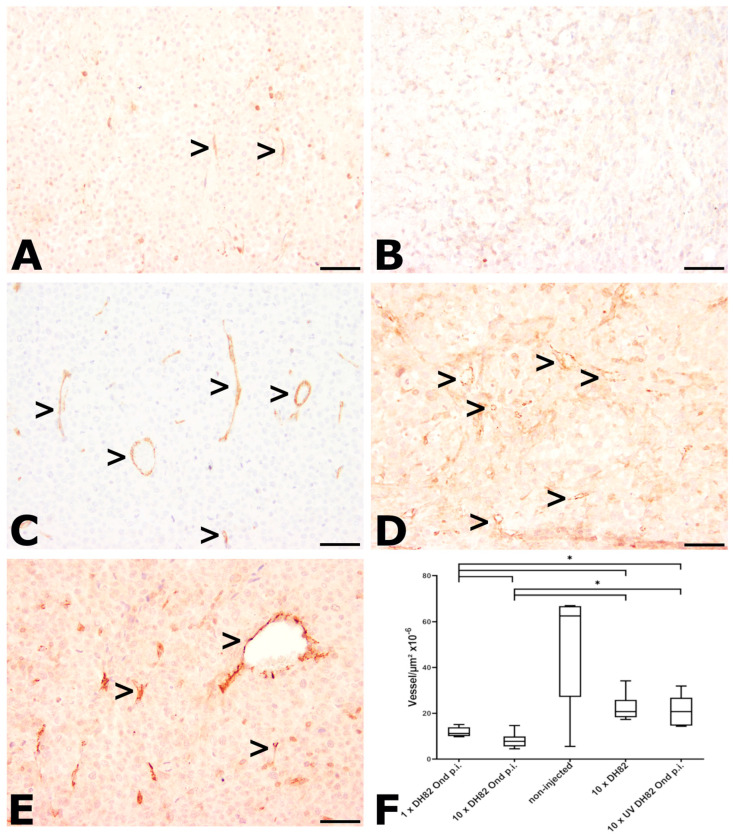
CD31 immunolabeling at 77 dpt for determination of the intratumoral vessel density. Since DH82 cells can also express CD31, only CD31-positive structures forming a distinct lumen were counted as vessels. (**A**) 1× DH82 Ond p.i. tumors and (**B**) 10× DH82 Ond p.i. neoplasms develop few vessels, with some localizations lacking any vessels. (**C**) Non-injected controls showed numerous vessels, while (**D**) 10× DH82 tumors and (**E**) 10× UV DH82 Ond p.i. tumors exhibited moderate numbers of vessels (>). (**F**) Graphical display of the vessel density within the different groups. 1× DH82 Ond p.i.: tumors after a single intratumoral injection (1× DH82 Ond p.i.) of persistently canine distemper virus strain Onderstepoort-infected DH82 cells (n = 6); 10× DH82 Ond p.i.: tumors after ten intratumoral injections of DH82 Ond p.i. cells (n = 6); 10× DH82: tumors after ten intratumoral injections of DH82 cells (n = 6); 10× UV DH82 Ond p.i.: tumors after ten intratumoral injections of UV-inactivated, homogenized DH82 Ond p.i. cells (n = 5); non-injected: tumors that did not receive any injection (n = 5). * *p* < 0.05. Scale bar: 100 µm; scale bar of insert: 20 µm.

Intratumoral vessel density was investigated by using the antibody CD31. Since DH82 cells also express CD31 [13], the vessel density was determined by counting all CD31-positive structures, which formed a distinct lumen in 3.975 mm^2^ (25 high-power fields (HPFs) or in the whole tumor area if the tumor was smaller than 3.975 mm^2^; Figure 5). At both time points, the 10× DH82 Ond p.i. group showed the lowest median (5.9 × 10^−6^ vessels/µm^2^ at 63 dpt; 11.2 × 10^−6^ vessels/µm^2^ at 77 dpt). The highest median of all groups at both time points was observed in the non-injected controls (134.9 × 10^−6^ vessels/µm^2^ at 63 dpt; 62.5 × 10^−6^ vessels/µm^2^ at 77 dpt). At 63 dpt, the 10× DH82 Ond p.i. tumors displayed a significantly lower vessel density than the 10× DH82 neoplasms and non-injected control tumors. Furthermore, the non-injected neoplasms showed a higher intratumoral vessel density than the 10× DH82 and 10× UV-DH82 Ond p.i. neoplasms. At 77 dpt, the 10× DH82 Ond p.i. tumors presented with a lower vessel density than all other groups except the non-injected controls. Additionally, the 1× DH82 Ond p.i. neoplasms displayed a significantly lower vessel density than the 10× DH82 and 10× UV DH82 Ond p.i. tumors (Figure 5F).

#### 2.2.5. Intratumoral Infiltration with Murine Macrophages

**Figure 6:**
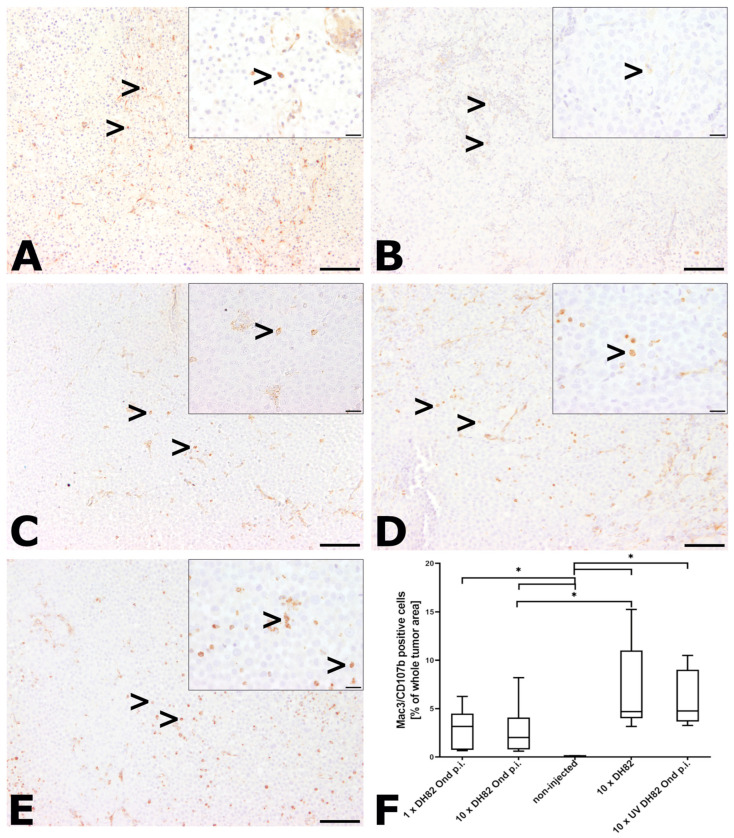
Mac3/CD107b immunolabeling at 77 dpt. Positive cells show a coarse cytoplasmic staining (>). (**A**) 1× DH82 Ond p.i.; (**B**) 10× DH82 Ond p.i. tumor; (**C**) non-injected tumor; (**D**) 10× DH82 tumors; (**E**) 10× UV DH82 Ond p.i. tumor. (**F**) Graphical display of the percentage of Mac3/CD107b positive cells in the different groups. Non-infected tumors showed less Mac3/CD107b positive cells than all other groups at 77 dpt. The 10× DH82 tumors showed higher numbers of Mac3/CD107b positive cells than the 10× DH82 Ond p.i. tumors. 1× DH82 Ond p.i.: tumors after a single intratumoral injection (1× DH82 Ond p.i.) of persistently canine distemper virus strain Onderstepoort-infected DH82 cells (n = 6); 10× DH82 Ond p.i.: tumors after ten intratumoral injections of DH82 Ond p.i. cells (n = 6); 10× DH82: tumors after ten intratumoral injections of DH82 cells (n = 6); 10× UV DH82 Ond p.i.: tumors after ten intratumoral injections of UV-inactivated, homogenized DH82 Ond p.i. cells (n = 5); non-injected: tumors that did not receive any injection (n = 5). * *p* < 0.05. Scale bar: 100 µm; scale bar of insert: 20 µm.

The percentage of intratumoral murine macrophages of all cells within the tumor area was evaluated using an antibody directed against Mac3/CD107b (Figure 6). Necrotic areas were excluded from the analysis. The percentage of Mac3/CD107b immunoreactive cells ranged in all groups and time points from 0.01% to 16.17%. The non-injected controls exhibited the lowest median number of all groups (0.05% at 63 dpt; 0.08% at 77 dpt) and showed significantly lower numbers than all other groups at both time points. At 63 dpt, the highest median percentage was observed in the 10× DH82 neoplasms (6.86%). At 77 dpt, the 10× UV-DH82 Ond p.i. neoplasms displayed the highest median percentage (4.76%). Additionally, at 77 dpt, the 10× DH82 Ond p.i. tumors showed significantly lower percentages of Mac3/CD107b immunoreactive cells than the 10× DH82 group (Figure 6F).

#### 2.2.6. Immunohistological Detection of CDV Nucleoprotein

**Figure 7:**
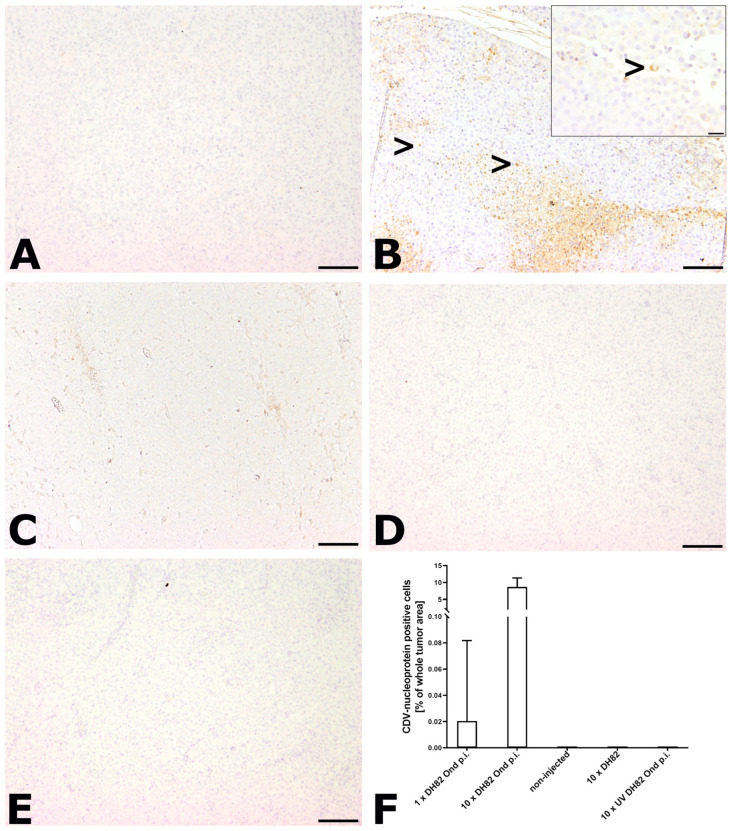
Canine distemper virus (CDV)-nucleoprotein immunolabeling at 77 dpt. (**A**) The 1× DH82 Ond p.i. tumors lacked immunoreactivity. (**B**) The 10× DH82 Ond p.i. neoplasms displayed multifocal CDV nucleoprotein positive cells (>). (**C**) Non-injected tumors, (**D**) 10 DH82 neoplasms, and (**E**) UV DH82 Ond p.i. lacked immunolabeling for CDV nucleoprotein. (**F**) Graphical display of the percentage of CDV nucleoprotein positive cells in the different groups. 1× DH82 Ond p.i.: tumors after a single intratumoral injection (1× DH82 Ond p.i.) of persistently canine distemper virus strain Onderstepoort-infected DH82 cells (n = 6); 10× DH82 Ond p.i.: tumors after ten intratumoral injections of DH82 Ond p.i. cells (n = 6); 10× DH82: tumors after ten intratumoral injections of DH82 cells (n = 6); 10× UV DH82 Ond p.i.: tumors after ten intratumoral injections of UV-inactivated, homogenized DH82 Ond p.i. cells (n = 5); non-injected: tumors that did not receive any injection (n = 5). Scale bar: 100 µm; scale bar of insert: 20 µm.

The percentage of CDV nucleoprotein positive cells was calculated by counting all tumor cells exhibiting a granular to coarse cytoplasmic, sometimes membrane-associated brownish staining, divided by the total number of cells (Figure 7). Necrotic areas were excluded from the analysis. The control groups (non-injected, 10× DH82 cells, 10× UV DH82 Ond p.i. cells) did not show any positive cells at any time point. Within the 1× DH82 Ond p.i. group at 77 dpt, one animal showed CDV nucleoprotein positive cells (0.08% of whole tumor area), whereas no CDV nucleoprotein was detected in all other animals of this group. Within the 10× DH82 Ond p.i. group, all tumors showed positive cells at 63 dpt, ranging from 3.05% to 11.15% of all cells (median: 6.73%). At 77 dpt, the number of infected cells in four animals ranged between 0.10% and 11.32%, while two neoplasms displayed no positive cells (group median: 1.25%). There was no significant difference regarding the percentage of CDV nucleoprotein positive cells between the single-injected and the tenfold-injected groups (Figure 7F).

### 2.3. RT-qPCR for Detection of CDV mRNA Transcripts

**Figure 8:**
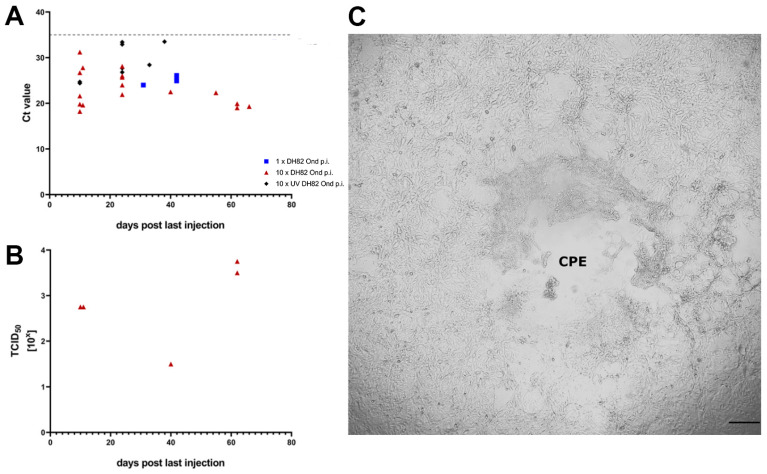
(**A**) Cycle threshold (Ct) values for CDV nucleoprotein RNA transcripts in tumor tissue injected once (blue squares) or 10 times (red triangles) intratumorally with persistently canine distemper virus strain Onderstepoort (CDV-Ond)-infected DH82 cells (DH82 Ond p.i.) or 10 times with UV-inactivated homogenized DH82 Ond p.i. (UV DH82 Ond p.i.; black rhombs) cells at different time points after the last injection. The dotted grey line represents the detection limit. (**B**) TCID50 values of CDV isolated from tumor tissue injected 10 times with DH82 Ond p.i at different time points after the last injection. (**C**) Virus titration of tumor tissue injected 10 times with DH82 Ond p.i. cells. Titration of homogenized tumor material on Vero.dogSLAM cells resulted in a variable cytopathic effect (CPE). Dilution: 10^−3^; scale bar: 100 µm.

CDV mRNA transcripts were determined in the tumor tissue injected once or ten times with DH82 Ond p.i. cells or ten times with UV-DH82 Ond p.i. cells (Figure 8A). In 31 of 37 investigated tumors, viral mRNA transcripts were detected. The overall CT values ranged from 18.2 (37 999.98 copies/ng RNA; tenfold injection with DH82 Ond p.i. cells, 63 dpt) to 33.5 (7.70 copies/ng RNA; tenfold injection with UV DH82 Ond p.i. cells, 91 dpt). In all tumors (100%) injected either once or tenfold with DH82 Ond p.i. cells, viral mRNA transcripts were present, with the latest detection 66 days after the last injection (119 dpt). In the tumors injected ten times with UV-DH82 Ond p.i. cells, eight of fourteen (57.14%) animals presented with a positive result in RT-qPCR. An overview of all CT values, copy numbers, and time points after the last intratumoral injection is given in Supplementary Table S3.

### 2.4. Virus Titration

For the detection of infectious virus, a virus titration of homogenized, frozen tumor tissue of animals of the 1× DH82 Ond p.i., the 10× DH82 Ond p.i., and the 10× UV DH82 Ond p.i. groups was performed. Since the first dilution (10^−1^) was highly viscous, the evaluation of this dilution was very limited. Therefore, only dilutions starting from 10^−2^ were taken into account. Neither the 10× UV DH82 Ond p.i. tumors nor the 1× DH82 Ond p.i. tumors showed a cytopathic effect (CPE) in the analyzed dilutions. Six out of seventeen (35.29%) animals of the 10× DH82 Ond p.i. group presented with a CPE (Figure 8B,C). Interestingly, the latest time point at which a CPE was observed was 62 days (115 dpt) after the last intratumoral injection. An overview of all titers and time points after the last intratumoral injection is given in Supplementary Table S3. Using immunofluorescence to detect CDV nucleoprotein subsequently to the virus titration, an association of the CPE observed in the virus titration with an actual CDV infection was confirmed.

## 3. Discussion

The aim of this study was to investigate the influence of the intratumoral application of persistently CDV-infected canine histiocytic sarcoma cells into established xenotransplanted canine histiocytic sarcomas in a murine model. In previous publications, subcutaneous xenotransplantation of DH82 cells presented a successful and suitable model for oncolytic studies [13,27]. Furthermore, a xenotransplantation of persistently infected DH82 cells led to an initial tumor growth, followed by a constant regression starting at 14 dpt [13,28]. In a study by Armando et al. (2021), an acute CDV infection of DH82 xenotransplants resulted in a transient stagnation of tumor growth but without reaching complete regression [27]. Thus, it was hypothesized that a combination of the already established DH82 xenotransplantation model treated intratumorally with persistently CDV-infected DH82 cells as a permanent source of virus would lead to a tumor regression. In the current study, the 10× DH82 Ond p.i. tumors exhibited a smaller relative tumor volume than all or most other groups from 56 dpt to 77 dpt. The oncolytic effect of CDV was also shown by the detection of increased areas of necrosis, which was higher in this group than in the 10× DH82 tumors at 63 dpt as well as in the 10× UV DH82 Ond p.i. groups at 77 dpt and in the non-injected tumors at both time points. However, based on the small animal number per group, inter-individual differences cannot be excluded, and the data need to be interpreted with caution.

While in the present study a transient stagnation of tumor growth was achieved, previous investigations report a complete tumor regression of different tumors after a single intratumoral injection with the Western Reserve strain of Vaccinia virus [29] as well as with a GM-CSF-armed strain of the same virus [30]. In both of these studies, immunocompetent hosts were used, and the authors attribute part of the success to the antitumoral immune answer. In the present study, *scid* mice were used. Therefore, it might be possible that the antitumoral effect of the applied CDV might be enhanced in hosts with a competent immune system. On the other hand, an enhanced viral clearance and, thus, a reduced tumoricidal effect in immunocompetent patients cannot be excluded. After a single injection with DH82 Ond p.i. cells, the tumors showed promising results regarding the tumor volume as well as the histological and immunohistological parameters. However, in most aspects, the tenfold injection with the same cells seemed to be more effective. This might be not only due to the higher viral load but also to the fractionated application. This has also been shown in another work, in which rhabdomyosarcomas in a murine model were treated with an attenuated Herpes simplex virus (NV1020, NV1066) with the same dose but either injected as a single application or as multiple injections [31]. With the latter therapeutic approach, an improved viral distribution and, thus, complete regressions could be achieved [31].

At 77 dpt, four out of six (66.67%) of the 10× DH82 Ond p.i. tumors showed CDV nucleoprotein positive tumor cells. In contrast, CDV nucleoprotein positive cells were found in only one animal that received a single injection with the same cells at the same time point. Armando et al. (2021) mention that less than 0.5% of the whole tumor area was positive for CDV nucleoprotein after a tenfold acute infection of canine histiocytic sarcoma xenografts [27]. Since, in the present study, eight out of eleven animals of the 10× DH82 Ond p.i. group showed values over 1% (two animals even exceeding 10%), the application of persistently infected cells seems to be superior to the acute infection concerning viral distribution in this model. In a previous study, DH82 Ond p.i. cell xenotransplants showed a complete regression after an initial phase of growth. In this study, approximately 100% of the tumor cells were CDV-infected [28]. This indicates that, after a tenfold DH82 Ond p.i. cell injection, the number of infected cells is still too small to result in definitive regression in this model. According to Boisgerault et al. [32], the viral particle-to-cell ratio is crucial when it comes to a permanent regression. The authors of this previous study [32] achieved improved results after multiple intratumoral virus applications, with up to 22 days in between the applications; a prolonged application of the tenfold DH82 Ond p.i. cell injections could enhance the oncolytic effect in our model.

Tumors of a certain size (1–2 mm in diameter) depend on their own vasculature for an appropriate growth and supply [10]; thus, intratumoral vessels represent an interesting target for oncolytic therapy. Armando et al. (2020) reported an increased oxidative stress and a reduced HIF-1α angiogenic pathway in DH82 Ond p.i. cells in contrast to non-infected DH82 cells in vitro [33]. Those results were confirmed in an in vivo experiment, in which an acute CDV infection of canine histiocytic sarcoma cells as well as a xenotransplantation of persistently CDV-infected DH82 cells led to a reduced intratumoral vessel density [13,27]. In the present study, the angiostatic effect of an intratumoral injection of persistently CDV-infected DH82 cells in established xenotransplants was confirmed. The 10× DH82 Ond p.i. tumors exhibited a significantly lower vessel density than the groups without CDV infection at 63 dpt. At 77 dpt, the 10× DH82 Ond p.i. tumors showed a significantly lower vessel density than the 10× DH82 and 10× UV-DH82 Ond p.i. tumors as well as the 1× DH82 Ond p.i. neoplasms.

The mitotic rate, which was lower in the once or tenfold DH82 Ond p.i. cell-injected tumors than two of the control groups at 77 dpt, proves that the smaller tumor volume is not only caused by necrosis but also by a lower growth rate. Similar results were achieved in a study with mammary adenocarcinoma xenografts infected with the attenuated vaccine strain CDV-L, where mice received four intratumoral applications of CDV-L over 12 days. This led to a tumor growth restriction without infection-associated toxicity to vital organs [34]. Additionally, a study by Pfankuche et al. (2017) demonstrated a lower mitotic rate in DH82 Ond p.i. cell xenotransplants compared to non-infected controls [13].

Previous studies suggest an increased apoptotic rate in different tumor types after treatment with oncolytic viruses [34,35]. In contrast to these findings, a low apoptotic rate was demonstrated in the present study, independent of the analyzed group. Most animals showed values of less than 1% apoptotic cells, which renders the importance of this pathogenetic mechanism in the current study design questionable. Similar findings regarding the minor importance of apoptosis in CDV Ond-infected DH82 cell xenografts were obtained in a previous study [27]. Taken together, apoptosis seems to play a minor biological role in CDV-mediated viral oncolysis.

Tumor-associated macrophages (TAMs) play an important role in modulation of the tumor microenvironment [36]. Prominent histiocytic infiltrations have been noticed both in spontaneous and xenotransplanted canine histiocytic sarcomas [37]. There were no elevated numbers of murine macrophages observed in tumors injected with DH82 Ond p.i. cells. Nevertheless, TAMs can occur in different polarizations, making elevated numbers of intratumoral macrophages not necessarily a sign of a tumoricidal microenvironment. While the classically activated M1 macrophages are known to have antitumoral properties, alternatively activated M2 macrophages may produce various growth factors and, thus, promote tumor growth [38,39]. Since elevated numbers of macrophages in association with tumor growth stagnation and regression have been observed in previous studies [28,37], the present model and application scheme could be combined with using a modified CDV, which delivers a functional granulocyte–macrophage colony-stimulating factor (GM-CSF) for an enhanced effect [40].

Surprisingly, although the growth arrest of the persistently infected tumors was only transient, viral mRNA transcripts were detected in all tumors injected either once or tenfold with DH82 Ond p.i. cells, with the latest time point 66 days after the last injection. In contrast to the positive results of the qPCR, replicative virus was not detected in all of those animals via virus titration. Nevertheless, the latest time point at which a CPE could be observed was 62 days after the last injection in the 10× DH82 Ond p.i. group. The virus titration results showed that there is a long-term detectable effect using a persistent CDV Ond infection in contrast to an acute one, making the first one a promising approach. Previous studies proved a complete regression of DH82 Ond p.i. cell xenotransplants [13,28]. A possible explanation for the decreased oncolytic effect with infectious virus still detectable within the tumors might be a regression of the injected DH82 Ond p.i. cells, resulting in an unfavorable ratio of infected versus non-infected tumor cells. Thus, a prolonged or repeated treatment cycle might be indicated, which could lead to elevated relative numbers of persistently infected cells within the tumors.

Despite the promising results, this study faces several limitations. The number of animals (n = 6) per group and time point was relatively low, and especially at later time points, some animals had to be preliminarily sacrificed for human reasons, which further reduced the group size. In addition, inter-individual variations contributing to the results cannot be excluded. Furthermore, it should be taken into account that immunocompromised mice (*scid* mice) were used, rendering the effect of the adaptive immune system in spontaneous clinical cases unpredictable.

The results of the present study confirmed the oncolytic potential of CDV and showed, compared to previous studies [27], that a persistent infection is superior to an acute one. In addition to the alteration of the application regime, resulting in multiple applications, the usage of genetically modified viruses could be a complementary modification to enhance the oncolytic effect for CDV as described by others [15,41,42]. To enhance the angiostatic effect observed in the present study, a CDV-expressing vasostatin, as described in vitro [43], would represent an ideal candidate for such an approach. A similar design was presented by Mullen et al. (2024) by using a modified Herpes simplex virus expressing endostatin that resulted in decreased numbers of intratumoral blood vessels as well as a complete necrosis of some of the treated tumors in a murine model of human colon carcinoma [42]. Overall, the present study shows a promising approach, which could be adjusted in different ways in future works to achieve complete and lasting regressions of canine histiocytic sarcomas.

## 4. Materials and Methods

### 4.1. Cell Culture

The permanent DH82 cell line was obtained from the European Collection of Authenticated Cell Cultures (ECACC No. 94062922). The persistently CDV Ond-infected DH82 cells (DH82 Ond p.i.) used in this experiment were produced as described elsewhere [26]. Both cell populations were cultivated according to standard protocols [13,26] using Eagle’s minimal essential medium with Earle’s salts (MEM, Gibco, Thermo Fisher Scientific, Roskilde, Denmark) containing 10% fetal bovine serum (Capricorn Scientific, Ebsdorfergrund, Germany), 1% penicillin/streptomycin (Sigma-Aldrich Chemie GmbH, Taufkirchen, Germany), as well as 1% non-essential amino acids (Sigma-Aldrich Chemie GmbH). For UV-inactivation of DH82 Ond p.i. cells, cells were passaged and transferred to sterile cell culture dishes (Nunclon^®^, Nunc A/S, Thermo Fisher Scientific) with a concentration of 3 × 10^6^/100 µL serum-free medium (176.37 µL/cm^2^). Those were placed under UV light (Osram HNS 15 Watt G13, OSRAM GmbH, Munich, Germany) for 8 h. Following UV irradiation, the cell culture dishes were scraped, and the cell suspension was transferred into collection tubes. The evaporated medium was measured and replaced by Eagle’s minimal essential medium with Earle’s salts with 1% penicillin/streptomycin and 1% non-essential amino acids. The obtained suspension was physically homogenized (Omni PCR Tissue Homogenizing Kit, Omni International, Inc., Warrenton, VI, USA). To confirm the inactivity of the virus, a virus titration was performed (see below). Furthermore, parts of the suspension were cultivated again for seven days to validate the cell destruction.

### 4.2. Xenotransplantational Mouse Model

Approval for the animal experiment was given by the authorities of lower Saxony (Landesamt für Verbraucherschutz und Lebensmittelsicherheit, LAVES; file number: 33.8-42502-04-16/2141).

Six commercially bred female severe combined immunodeficient (*scid*) mice (CB17/lcr-Prkd^scid^/lcrlcoCrl) per group and time point were obtained (Charles River Laboratories, Sulzfeld, Germany) at an age of four to five weeks. The six mice per group were randomized, and each group was kept in an individually ventilated cage (Tecniplast Deutschland GmbH, Hohenpeißenberg, Germany). Autoclaved water as well as total pathogen-free maintenance diet pellets (Altromin Sepzialfutter GmbH & Co. KG, Lage, Germany) were available ad libitum. The mice were obtained two weeks prior to the xenotransplantation. During these two adaptation weeks, they were familiarized with handling procedures such as weighing.

For xenotransplantation, an injection of 3.0 × 10^6^ DH82 cells suspended in 100 µL serum-free MEM medium into the subcutis of the left flank was performed using single-use pen needles (Omnican F, B. Braun, Melsungen, Germany). After transplantation, a period of 35 days followed, in which the body weight of the mice and the tumor growth were measured every second to third day. For measuring the tumor diameter, a Vernier precision caliper was used. According to Grote et al. (2001), the tumor volume was calculated with the following formula: (shortest diameter^2^ × longest diameter)/2 [20].

### 4.3. Tumor Treatment and Measurement

At 35 days after transplantation (35 dpt), when the tumors reached a median diameter of approximately 0.5 cm, intratumoral injections started. The animals either received one single injection with 3.0 × 10^6^ DH82 Ond p.i. cells (whole cells) in 100 µL medium or a tenfold injection each with 3.0 × 10^6^ DH82 Ond p.i. cells (whole cells) in 100 µL medium; 3.0 × 10^6^ DH82 cells (whole cells) in 100 µL medium; or 100 µL UV-inactivated, homogenized DH82 Ond p.i. (cell homogenate) cell suspension. The persistently CDV-infected DH82 cells, continuously producing infectious virus, were chosen as a permanent virus source. Those groups receiving ten intratumoral applications were injected every second day, starting at 35 dpt. Additionally, previously published data [27] from a group that received the same initial transplantation but was left untreated afterwards were included for comparison.

The animals were clinically scored every day by measuring tumor growth and body weight every second to third day until necropsy. Necropsies were performed at 77 dpt for all groups. There were two additional time points of necropsy (63 and 119 dpt) in the groups that were treated ten times. All mice with tumors showing a volume of more than 1.7 cm^3^ or an ulcerated tumor surface as well as those animals that reached a defined clinical score were preliminarily euthanized due to animal welfare reasons.

### 4.4. Necropsy and Sample Collection

At necropsy, tumor size was determined, and samples of the neoplasms were taken. The tumors were divided in at least two parts, with half of the tumor frozen at −80 °C in Tissue Tec^®^-O.C.T.^TM^ –compound (Sakura, Finetek USA, Inc., Torrance, CA, USA) and the other half fixed in 10% neutral-buffered formalin for further histological and immunohistological analyses (see Section 4.5). In addition to sampling of tumor tissue, a complete necropsy was performed, and representative samples of each organ system were taken for further histological examination.

### 4.5. Histological and Immunochistological Staining

After fixation for 24 h in 10% neutral-buffered formalin, the tissue samples were further processed and embedded in paraffin wax. A total of 120–200 serial sections of each tumor sample of approximately 2–3 µm thickness were prepared. Every 40th slide was stained with hematoxylin–eosin (HE). The most representative section was chosen for further histological analysis. The remaining serial sections were used for immunohistology using the avidin–biotin–peroxidase complex method (Vectastain^®^ Elite^®^ ABC Kit, BIOZOL Diagnosica Vertrieb GbmH, Eching, Germany) with 3,3′-diaminobenzidine tetrahydrochloride (Sigma-Aldrich Chemie GmbH) as a chromogen, as described elsewhere [23].

Supplementary Table S4 gives an overview of the antibodies used and their individual protocols.

### 4.6. Histological and Immunohistological Evaluation

Immunohistological and HE-stained slides were scanned with the Olympus VS200 slide scanner (Olympus Deutschland GmbH, Hamburg, Germany). For further analysis, QuPath version 0.3.2 was used [43]. The percentage of necrosis within the tumors was manually determined by marking the whole tumor area as well as the necrotic area (in µm^2^). The percentage was calculated by dividing the necrotic area by the whole tumor area and multiplying the result by 100. For the determination of immunopositive cells of the different stainings (CDV nucleoprotein, cleaved caspase 3, Mac3/CD107b), the tumor area was again manually marked. After that, a positive cell detection followed, and the ratio of positive to negative cells of all cells within the whole tumor area was measured. Thresholds were adjusted manually depending on the different staining intensities and the cell-associated distribution pattern in order to exclude unspecific background staining.

The mitotic rate and the vessel density were manually counted using a light microscope (Axiostar Plus, Carl Zeiss AG, Oberkochen, Germany). For the mitotic rate, the number of mitotic figures within 1.59 mm^2^ [10 high power fields (HPF, 400×)] in the tumor periphery was determined and divided by 10. For evaluating the vessel density, 25 randomly distributed HPFs were chosen, and all CD31-positive structures forming a distinct lumen were counted. In tumors smaller than 10 HPFs or 25 HPF, all mitotic figures or vessels, respectively, were counted. The vessel density was given as the number of vessels per µm^2^ tumor area.

### 4.7. RNA Isolation, Reverse Transcription (RT) and RT-qPCR

RNA isolation of the frozen tumor material, including DNA digestion with RNase- free DNase (Qiagen GmbH, Hilden, Germany), was performed using the RNeasy Mini Kit (Qiagen GmbH, Hilden, Germany) according to the manufacturer’s protocol. A GeneQuant pro (amersham plc, Amersham, UK) was utilized for spectroscopical measurement of the RNA concentration at 260 nm. Reverse transcription of the RNA and real-time quantitative PCR (RT-qPCR) were carried out as described before [26,44]. For the reverse transcription, Omniscript (Qiagen GmbH, Hilden, Germany), RNAse OUT (Invitrogen, Thermo Fisher Scientific), and Random Hexamers (Promega, Madison, WI, USA) were used. The reaction itself was performed in a Biometra Thermocycler T-Gradient ThermoBlock (American Laboratory Trading, East Lyme, CT, USA) with a temperature profile of 25 °C for 10 min, 37 °C for 1 h, and 93 °C for 5 min. The primers used for RT-qPCR were taken from the literature [MWG-Biotech, Ebersberg, Germany [26]] and diluted to a final concentration of 150 nM each. Furthermore, the Brilliant III Ultra-Fast SYBR^®^ Green QPCR Master Mix (Agilent Technologies, Santa Clara, CA, USA) was used. The reaction was performed with an AriaMx Real-time PCR System (Agilent Technologies) under the following conditions: one cycle of 3 min at 95 °C, 35 cycles of alternating 5 s at 95 °C and 10 s at 57 °C, one cycle of 30 s at 95 °C, 30 s at 65°C with a following stepwise increase in the temperature (0.5 °C for 5 s each until 95 °C was reached), and 30 s at 95 °C. Standard curves were generated using serially diluted RT-PCR amplicons (10^8^ to 10^2^ copies/µL). Two no-template controls were included in the reaction and served as negative controls.

### 4.8. Virus Titration

According to previous descriptions [45], virus titration was performed using mechanically homogenized tumor tissue. After centrifugation, the supernatant was diluted logarithmically from 10^−1^ to 10^−10^ in Dulbecco’s Modified Eagle’s Medium (DMEM, Gibco, Thermo Fisher Scientific) containing 1% penicillin/streptomycin (Sigma-Aldrich). For virus titration of the inactivated, homogenized DH82 p.i. cells used for treatment, the homogenized cell suspension was centrifuged, and the supernatant was diluted with the same medium from 10^−1^ to 10^−10^ dilution. We used 96-well titer plates (ThermoFischer Scientific) containing Vero.DogSLAM cells [1.5 × 10^4^ cells/well suspended in 100 µL DMEM/well with 10% fetal bovine serum (Capricorn Scientific), 1% penicillin/streptomycin (Sigma-Aldrich) and 0.1% phleomycin (Zeozin^®^, InvivoGen, Toulouse, France)]. The titer plates were incubated under standard conditions (37 °C, 5% CO_2_) for three days before the cytopathic effect was evaluated.

### 4.9. Immunofluorescence

Subsequent to the virus titration, an immunofluorescence for the detection of CDV nucleoprotein was performed in representative wells as previously described [34,43]. DH82 CDV Ond p.i. cells seeded at a density of 30,000 cells suspended in 100 µL culture medium per well served as positive controls. For the negative controls, the primary or secondary antibody, respectively, was omitted. After fixation with 4% buffered paraformaldehyde and permeabilization with 0.25% PBS-Titon X, a blocking with goat serum was performed, and the cells were incubated overnight at 4 °C with the primary antibody (1:100, CDV D110, A. Zurbriggen). After washing the cells with PBS with 1% Triton X, they were incubated with the secondary antibody (1:100, goat anti-mouse cyanine 3-conjugated, GaM-Cy3, Jackson ImmunoResearch Laboratories, Hamburg, Germany) for 2 h and counterstained with bisbenzimide (Sigma-Aldrich). The results were qualitatively evaluated using an Olympus IX-70 microscope (Olympus optical Co. GmbH, Hamburg, Germany) combined with an Olympus DP-72 camera. The Olympus CellSense standard software version 2.3 was used.

### 4.10. Statistical Evaluation

Statistical analyses were performed using SAS Enterprise Guide 7.1 (SAS Institute, Cary, NC, USA). Histological and immunohistological findings were evaluated using the Wilcoxon rank-sum test with the significance level *p* < 0.05. For designing graphs, GraphPad Prism 9.3.1 (GraphPad Software, San Diego, CA, USA) was used.

## Data Availability

All relevant data are included in the manuscript; the Supplementary Material can be obtained from the authors on reasonable request.

## Supplementary Materials

The following supporting information can be downloaded at: https://www.mdpi.com/article/10.3390/ijms25158297/s1.
