# Supplementary material for: Persistence of Infectious Canine Distemper Virus in Murine Xenotransplants of Canine Histiocytic Sarcoma Cells after Intratumoral Application
Source: Int J Mol Sci. 2024 Jul 30;25(15):8297. doi: 10.3390/ijms25158297 (PMC11311720; doi:10.3390/ijms25158297)
Supplement: Supplementary file 1 [file ijms-25-08297-s001.zip › ijms-3067230-supplementary.pdf]

International Journal of *Molecular Sciences*

MDPI

Article

Persistence of Infectious Canine Distemper Virus in Murine Xenotransplants of Canine Histiocytic Sarcoma Cells after Intratumoral Application

Mara Sophie Lombardo [1], Federico Armando [1,2], Katarzyna Marek [1], Karl Rohn [3], Wolfgang Baumgärtner [1] and Christina Puff [1,*]

[1] Department of Pathology, University of Veterinary Medicine Hannover, 30559 Hannover, Germany; Mara.Sophie.Lombardo@tiho-hannover.de (M.S.L.); Federico.Armando@unipr.it (F.A.); Wolfgang.Baumgaertner@tiho-hannover.de (W.B.); Christina.Puff@tiho-hannover.de (C.P.)
[2] University of Parma, Veterinary Medicine Department, Pathology Unit, Parma, Italy
[3] Institute for Biometry, Epidemiology and Information Processing, University of Veterinary Medicine Hannover, 30559 Hannover, Germany; Karl.Rohn@tiho-hannover.de
* Correspondence: Christina.Puff@tiho-hannover.de

Supplementary material:

This file includes:

Tables: 4

Citation: To be added by editorial staff during production.

Academic Editor: Firstname Lastname

Received: date
Revised: date
Accepted: date
Published: date

Copyright: © 2023 by the authors. Submitted for possible open access publication under the terms and conditions of the Creative Commons Attribution (CC BY) license (https://creativecommons.org/licenses/by/4.0/).

Supplementary table S1: Overview of significant differences of percentage tumor volume.

**Investigated time point**	**Group**	**Groups with significantly larger relative tumor volumes**
43 dpt	1 x DH82 Ond p.i. cells	• 10 x UV-DH82 Ond p.i. cells<br>• Non-injected
	10 x DH82 Ond p.i. cells	-
	10 x DH82 cells	• Non-injected
	10 x UV DH82 Ond p.i. cells	-
	Non-injected	-
49 dpt	1 x DH82 Ond p.i. cells	• 10 x UV-DH82 Ond p.i. cells<br>• Non-injected
	10 x DH82 Ond p.i. cells	• 10 x UV-DH82 Ond p.i. cells<br>• Non-injected
	10 x DH82 cells	• 10 x UV-DH82 Ond p.i. cells<br>• Non-injected
	10 x UV DH82 Ond p.i. cells	-
	Non-injected	-
56/57 dpt	1 x DH82 Ond p.i. cells	-
	10 x DH82 Ond p.i. cells	• 1 x DH82 Ond p.i. cells<br>• 10 x UV-DH82 Ond p.i. cells<br>• Non-injected
	10 x DH82 cells	• 1 x DH82 Ond p.i. cells<br>• 10 x UV-DH82 Ond p.i. cells<br>• Non-injected
	10 x UV DH82 Ond p.i. cells	• Non-injected
	Non-injected	-
63/64 dpt	1 x DH82 Ond p.i. cells	• Non-injected
	10 x DH82 Ond p.i. cells	• 1 x DH82 Ond p.i. cells<br>• 10 x UV-DH82 Ond p.i. cells<br>• Non-injected

Supplementary table S1 continued.

Investigated time point	Group	Groups with significantly larger relative tumor volumes
63/64 dpt continued	10 x DH82 cells	• 10 x UV-DH82 Ond p.i. cells<br>• non-injected
	10 x UV DH82 Ond p.i. cells	-
	Non-injected	• 10 x UV-DH82 Ond p.i. cells
71 dpt	1 x DH82 Ond p.i. cells	-
	10 x DH82 Ond p.i. cells	• 1 x DH82 Ond p.i. cells<br>• 10 x DH82 cells<br>• 10x UV-DH82 Ond p.i. cells<br>• non-injected
	10 x DH82 cells	• 1 x DH82 Ond p.i. cells<br>• non-injected
	10 x UV DH82 Ond p.i. cells	• 1 x DH82 Ond p.i. cells<br>• non-injected
	Non-injected	-
76/77 dpt	1 x DH82 Ond p.i. cells	-
	10 x DH82 Ond p.i. cells	• 1 x DH82 Ond p.i. cells<br>• 10 x DH82 cells<br>• 10x UV-DH82 Ond p.i. cells<br>• non-injected
	10 x DH82 cells	• 1 x DH82 Ond p.i. cells
	10 x UV DH82 Ond p.i. cells	• non-injected
	Non-injected	-
84 dpt	10 x DH82 Ond p.i. cells	-
	10 x DH82 cells	-
	10 x UV DH82 Ond p.i. cells	-
90 dpt	10 x DH82 Ond p.i. cells	-

Supplementary table S1 continued

Investigated time point	Group	Groups with significantly larger relative tumor volumes
90 dpt continued	10 x DH82 cells	-
	10 x UV DH82 Ond p.i. cells	-

dpt: days post transplantation; DH82 Ond p.i. cells: persistently canine distemper virus strain Onderstepoort infected DH82 cells; -: no data available of this group and time point

Supplementary table S2: Overview of percentage of intratumoral area of necrosis, mitotic rate, percentage of intratumoral CDV nucleoprotein positive cells, intratumoral vessel density, intratumoral apoptotic rate and intratumoral murine macrophages at 63 dpt and 77 dpt. Median (minimum; maximum)

Parameter	Group	63 dpt	77 dpt
Necrosis (% of tumor area)	1 x DH82 Ond p.i. cells	-	42.61 (0.26; 96.13)
	10 x DH82 Ond p.i. cells	29.19 (13.20; 72.08)	70.71 (15.21; 89.81)
	10 x DH82 cells	6.06 (0.00; 37.74)	16.82 (0.00; 82.84)
	10 x UV DH82 Ond p.i. cells	13.69 (10.45; 15.10)	2.98 (0.26; 42.43)
	Non-injected	0.49 (0.04; 0.93)	14.86 (1.46; 33.49)
Mitotic rate (mean of 10 x 0.159 mm²)	1 x DH82 Ond p.i. cells	-	2.4 (0,0; 9.7)
	10 x DH82 Ond p.i. cells	6.1 (5.5; 9.7)	0.8 (0.0; 2.0)
	10 x DH82 cells	11.7 (9.6; 15.3)	8.6 (0.0; 11.1)
	10 x UV DH82 Ond p.i. cells	0.2 (0.0; 6.2)	8.5 (7.3; 8.8)
	Non-injected	7.2 (5.1; 9.6)	6.5 (5.0; 9.0)
CDV nucleoprotein (% of tumor cells)	1 x DH82 Ond p.i. cells	-	0.00 (0.00; 0.08)
	10 x DH82 Ond p.i. cells	6.72 (3.05; 11.15)	1.25 (0.00; 11.32)
	10 x DH82 cells	0.00 (0.00; 0.00)	0.00 (0.00; 0.00)
	10 x UV DH82 Ond p.i. cells	0.00 (0.00; 0.00)	0.00 (0.00; 0.00)

Supplementary table S2 continued.

Parameter	Group	63 dpt	77 dpt
CDV nucleoprotein (% of tumor cells) continued	Non-injected	0.00 (0.00; 0.00)	0.00 (0.00; 0.00)
Vessel density (x10^{-6} vessels/µm^2)	1 x DH82 Ond p.i. cells	-	11.19 (9.81; 15.09)
	10 x DH82 Ond p.i. cells	5.90 (2.62; 11.06)	7.82 (4.53; 14.68)
	10 x DH82 cells	21.62 (15.59; 29.16)	20.75 (17.35; 34.19)
	10 x UV DH82 Ond p.i. cells	18.53 (3.14; 27.85)	20.72 (14.33; 31.93)
	Non-injected	134.88 (110.75; 153.25)	62.50 (5.56; 66.75)
Apoptotic rate (% of cleaved caspase 3 immunoreactive cells of tumor cells)	1 x DH82 Ond p.i. cells	-	0.17 (0.04; 1.77)
	10 x DH82 Ond p.i. cells	0.11 (0.06; 0.22)	0.02 (0.00; 0.19)
	10 x DH82 cells	0.20 (0.05; 0.33)	0.08 (0.04; 1.00)
	10 x UV DH82 Ond p.i. cells	0.19 (0.06; 1.13)	0.25 (0.13; 0.41)
	Non-injected	0.11 (0.02; 0.90)	0.18 (0.12; 0.31)
Murine macrophages (% of Mac3/CD107b positive cells of tumor cells)	1 x DH82 Ond p.i. cells	-	3.17 (0.66; 6.27)
	10 x DH82 Ond p.i. cells	6.63 (3.52; 16.17)	2.02 (0.61; 8.21)
	10 x DH82 cells	6.86 (1.15; 7.15)	4.69 (3.15; 15.25)
	10 x UV DH82 Ond p.i. cells	5.64 (2.77; 8.59)	4.76 (3.26; 10.50)
	Non-injected	0.05 (0.01; 0.27)	0.08 (0.03; 0.15)

dpt: days post transplantation; DH82 Ond p.i. cells: persistently canine distemper virus strain Onderstepoort infected DH82 cells; HPF: high power field; -: no data available of this group and time point

Supplementary table S3: Overview of individual CT-values and $TCID_{50}$.

Group	Animal	Days since last injection	CT-value	Number of copies / ng RNA	$TCID_{50}$ (excl. 10^{-1} dilution)
1 x DH82 Ond p.i. cells, planned necropsy at 77 dpt	1	42	26.1	174.42	No CPE
	2	31*	24.0	737.20	No CPE
	3	42	25.0	359.01	No CPE
	4	42	24.9	393.30	No CPE
	5	42	25.5	260.30	No CPE
	6	42	25.7	228.00	No CPE
10 x DH82 Ond p.i. cells, planned necropsy at 63 dpt	1	10	19.8	12 787.00	$10^{2.75}$
	3	10	26.7	109.63	No CPE
	4	10	31.2	5.00	No CPE
	5	10	21.6	3 838.00	No CPE
	6	10	18.2	37 999.98	$10^{2.75}$
10 x DH82 Ond p.i. cells, planned necropsy at 77 dpt	1	24	24.0	742.90	No CPE
	2	24	21.9	3 021.00	No CPE
	3	24	26.0	180.50	No CPE
	4	24	28.1	44.27	No CPE
	5	11*	27.8	53.96	No CPE
	6	24	25.7	218.50	No CPE
10 x DH82 Ond p.i. cells, planned necropsy at 119 dpt	1	62*	19.9	11 951.00	$10^{3.5}$
	2	55*	22.3	2 299.00	No CPE
	3	66	19.3	18 582.00	No CPE

Supplementary table S3 continued.

Group	Animal	Days since last injection	CT-value	Number of copies / ng RNA	$TCID_{50}$ (excl. 10^{-1} dilution)
10 x DH82 Ond p.i. cells, planned necropsy at 119 dpt continued	4	40*	22.5	1 976.00	$10^{1.5}$
	5	62*	19.0	22 040.00	$10^{3.75}$
	6	11*	19.6	17 917.00	$10^{2.75}$
10 x UV-DH82 Ond p.i. cells, planned necropsy at 63 dpt	1	10	No CT	No Cq	No CPE
	3	10	24.7	5 529.00	No CPE
	4	10	No CT	No Cq	No CPE
	5	10	24.4	6 669.00	No CPE
10 x UV-DH82 Ond p.i. cells, planned necropsy at 77 dpt	1	24	No CT	No Cq	No CPE
	2	24	26.8	1 225.50	No CPE
	3	24	32.9	14.44	No CPE
	4	24	No CT	No Cq	No CPE
	5	24	27.7	609.90	No CPE
	6	24	33.4	9.46	No CPE
10 x UV-DH82 Ond p.i. cells, planned necropsy at 119dpt	1	38*	33.5	7.70	No CPE
	2	33*	No CT	No Cq	No CPE
	3	33*	28.4	361.00	No CPE
	6	33*	No CT	No Cq	No CPE

dpt: days post transplantation; DH82 Ond p.i. cells: persistently canine distemper virus strain Onderstepoort infected DH82 cells; Cq: quantification cycle; CPE: cytopathic effect; *animal was preliminary sacrificed.

Supplementary table S4: Overview of used antibodies and their protocols.

Antigen	Name and origin of antibody, host species	Dilution	Pretreatment	Secondary antibody (biotinylated) and dilution	Positive control
CD44	CD44, E. Kremmer, rat	1:200	none	Rabbit anti rat 1:200	DH82 cell pellet
CDV-nucleoprotein	CDV D110, A. Zurbriggen, mouse	1:1000	MW + CB 20 min	Goat anti mouse 1:200	DH82 Ond p.i. cell pellet
Cleaved Caspase 3	Cleaved Caspase 3, Cell Signaling Technology (Danvers, MA, USA), rabbit	1:200	MW + CB 20 min	Goat anti rabbit 1:200	Spleen and lymph node of *scid* and immunocompetent mouse
CD31	CD31/ PECAM1, OriGene (Rockville, MD, USA), rabbit	1:800	MW + CB 20 min	Goat anti rabbit 1:200	Kidney of *scid* mouse
Mac3/ CD107b	Mac3/CD107 biotinylated, Bio-Rad (Hercules, CA, USA), rat	1:200	MW + CB 20 min	-	Spleen and lymph node of *scid* and immunocompetent mouse

CD: cluster of differentiation; CDV: canine distemper virus; PECAM: platelet endothelial cell adhesion molecule; MW + CB: incubation in microwave at 750 watt, covered with citrate buffer, pH 7.1; *scid*: severe combined immunodeficiency; -: no secondary antibody used (biotinylated first antibody).
